# Supplementary material for: Association between Dietary Salt Intake and Progression in the Gastric Precancerous Process
Source: Cancers (Basel). 2019 Apr 3;11(4):467. doi: 10.3390/cancers11040467 (PMC6520970; doi:10.3390/cancers11040467)
Supplement: Supplementary file 1 [file cancers-11-00467-s001.pdf]

Supplemental Materials

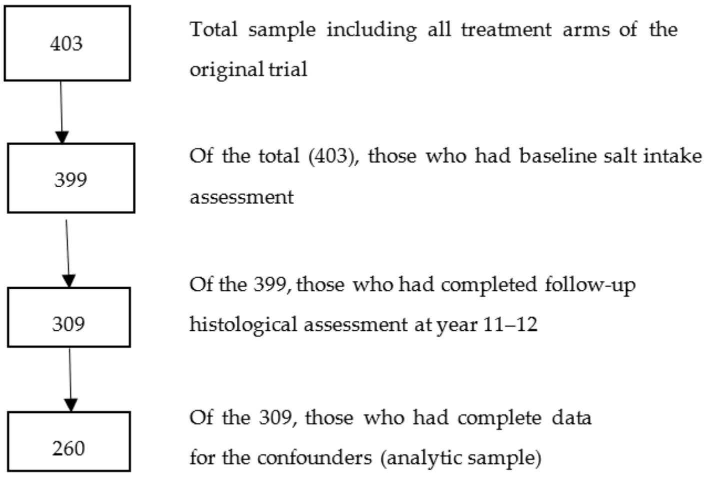

Figure S1. Sample Derivation Flowchart.

.

Table S1 Adjusted risk ratios for the association between salt intake at five months defined by urinary sodium/creatinine ratio and progression in the gastric precancerous process (11–12 years compared to five months).

	**Overall**		***H. pylori* +**		***H. pylori* -**		**Persistent *H. pylori* (Positive at Five Months and 11–12 years)**	
**Outcomes**	***n* with: *n* without Outcome**	**Risk Ratio (CI) *p*-Value [a]**	***n* with: *n* without Outcome**	**Risk Ratio (CI) *p*-Value [a]**	***n* with: *n* without Outcome**	**Risk Ratio (CI) *p*-Value [a]**	***n* with: *n* without Outcome**	**Risk Ratio (CI) *p*-Value [a]**
Overall progression in the gastric precancerous process Total *n* at risk =253	190:63	0.98 (0.90–1.07) $p = 0.61$	163:48	1.00 (0.91–1.09) $p = 0.94$	27:15	0.86 (0.66–1.11) $p = 0.24$	140:42	0.98 (0.89–1.07) $p = 0.62$
Incident progression to atrophic gastritis Total *n* at risk = 180	105:75	1.08 (0.97–1.21) $p = 0.15$	92:63	1.07 (0.95–1.19) $p = 0.26$	13:12	1.37 (0.91–2.08) $p = 0.13$	79:50	1.05 (0.93–1.17) $p = 0.43$
Incident progression to intestinal metaplasia Total *n* at risk = 198	90:108	1.01 (0.85–1.19) $p = 0.93$	80:88	1.01 (0.85–1.20) $p = 0.92$	10:20	1.15 (0.63–2.13) $p = 0.65$	69:74	1.00 (0.84–1.20) $p = 0.97$
Incident progression to dysplasia or gastric cancer Total *n* at risk =251	12:239	1.46 (1.11–1.93) $p = 0.007$	10:201	1.58 (1.19–2.10) $p = 0.002$	2:38	b	10:172	1.54 (1.16– 2.03) $p = 0.003$

[a] Adjusted for age, gender, car ownership, and fruit, vegetable intake. [b] Missing RR due to sparse data.

Table S2. Crude risk ratios for the association between salt intake at baseline, measured by urinary sodium creatinine ratio and progression in the gastric precancerous process (11–12 years compared to baseline).

**Outcomes**	**Overall**		***H. pylori* Positive at Five Months**		***H. pylori* Negative at Five Months [b]**		**Persistent *H. pylori* (Positive at Five Months and 11–12 years)**	
	***n* with: *n* without Outcome**	**Risk Ratio (CI) *p*-Value [a]**	***n* with: *n* without Outcome**	**Risk Ratio (CI) *p*-Value [a]**	***n* with: *n* without Outcome**	**Risk Ratio (CI) *p*-Value [a]**	***n* with: *n* without Outcome**	**Risk Ratio (CI) *p*-Value [a]**
Overall progression in the gastric precancerous process Total *n* at risk = 260	85:175	0.90 (0.73–1.09) $p = 0.28$	72:144	0.93 (0.75–1.16) $p = 0.53$	13:31	0.71 (0.47–1.08) $p = 0.11$	64:121	0.94 (0.75–1.17) $p = 0.58$
Incident progression to atrophic gastritis Total *n* at risk = 73	28:45	1.04 (0.81–1.33) $p = 0.77$	23:37	1.05 (0.83–1.34) $p = 0.67$	5:8	0.86 (0.4–1.86) $p = 0.70$	20:29	1.03 (0.79–1.34) $p = 0.82$
Incident progression to intestinal metaplasia Total *n* at risk = 175	63:112	0.94 (0.77–1.15) $p = 0.55$	56:89	0.95 (0.78–1.17) $p = 0.65$	7:23	0.82 (0.40–1.69) $p = 0.59$	51:73	0.99 (0.82–1.20) $p = 0.91$
Incident progression to dysplasia or gastric cancer Total *n* at risk = 253	10:243	1.17 (0.81–1.68) $p = 0.41$	8:203	1.35 (0.95–1.91) $p = 0.09$	2:40	0.36 (0.20–0.66) $p < 0.0001$	8:171	1.07 (0.81–1.43) $p = 0.63$

Table S3. Crude risk ratios for the association between salt intake at baseline, measured by sodium creatinine ratio and progression in the gastric precancerous process (11–12 years compared to five months).

Outcomes	Overall		*H. pylori* Positive at Five Months		*H. pylori* Negative at Five Months		Persistent *H. pylori* (Positive at Five Months and 11–12 years)	
	n with: *n* without Outcome	Risk Ratio (CI) *p*-Value [a]	*n* with: *n* without Outcome	Risk Ratio (CI) *p*-Value [a]	*n* with: *n* without Outcome	Risk Ratio (CI) *p*-Value [a,b]	*n* with: *n* without Outcome	Risk Ratio (CI) *p*-Value [a]
Overall progression in the gastric precancerous process Total *n* at risk = 253	190:63	0.94 (0.87–1.01) $p = 0.1$	163:48	0.97 (0.9–1.04) $p = 0.37$	27:15	0.8 (0.64–1.01) $p = 0.06$	140:42	0.95 (0.87–1.03) $p = 0.19$
Incident progression to atrophic gastritis Total *n* at risk =180	105:75	1.1 (0.99–1.22) $p = 0.07$	92:63	1.08 (0.97–1.21) $p = 0.14$	13:12	1.31 (0.87–1.95) $p = 0.19$	79:50	1.08 (0.97–1.2) $p = 0.17$
Incident progression to intestinal metaplasia Total *n* at risk =198	90:108	1.06 (0.91–1.23) $p = 0.43$	80:88	1.05 (0.9–1.23) $p = 0.53$	10:20	1.19 (0.75–1.88) $p = 0.47$	69:74	1.05 (0.9–1.23) $p = 0.53$
Incident progression to dysplasia or gastric cancer Total *n* at risk = 251	12:239	1.38 (1.00–1.91) $p = 0.05$	10:201	1.49 (1.08–2.07) $p = 0.02$	2:38	a	10:172	1.45 (1.05–2.00) $p = 0.02$

[a] Missing RR due to sparse data.

Table S4. Adjusted risk ratios for the association between salt intake at baseline, measured by the frequency of adding salt to foods, and progression in the gastric precancerous process (11–12 years compared to baseline).

Outcomes	Overall		*H. pylori* + at Five Months		*H. pylori* - at Five Months		Persistent *H. pylori* (Positive at Five Months and 11–12 years)	
	n with: *n* without Outcome	Risk Ratio (CI) *p*-Value [a]	*n* with: *n* without Outcome	Risk Ratio (CI) *p*-Value [a]	*n* with: *n* without Outcome	Risk Ratio (CI) *p*-Value [a]	*n* with: *n* without Outcome	Risk Ratio (CI) *p*-Value [a]
Overall progression in the gastric precancerous process Total *n* at risk =295	104:191	1.09 (0.85–1.4) $p = 0.50$	91:160	1.07 (0.78–1.45) $p = 0.68$	13:31	0.89 (0.50–1.57) $p = 0.69$	76:130	1.08 (0.81–1.44) $p = 0.58$
Incident progression to atrophic gastritis Total *n* at risk = 98	38:60	0.69 (0.46–1.04) $p = 0.07$	33:51	0.69 (0.39–1.21) $p = 0.2$	5:9	0.60 (0.18–2.03) $p = 0.41$	28:36	0.86 (0.53–1.42) $p = 0.56$
Incident progression to intestinal metaplasia Total *n* at risk = 203	71:132	1.08 (0.80–1.45) $p = 0.61$	64:109	1.13 (0.80–1.61) $p = 0.48$	7:23	1.86 (0.79–4.37) $p = 0.15$	57:84	1.08 (0.78–1.51) $p = 0.63$
Incident progression to dysplasia or gastric cancer Total *n* at risk = 288	12:276	1.07 (0.49–2.37) $p = 0.86$	10:236	2.59 (0.65–0.34) $p = 0.18$	2:40	b	9:192	1.38 (0.52–3.67) $p = 0.51$

[a] Adjusted for age, gender, car ownership, and fruit, vegetable intake. [b] Missing RR due to sparse data.

Table S5. Adjusted risk ratios for the association between salt intake at baseline, measured as **total salt added to food**, and progression in the gastric precancerous process (11–12 years compared to baseline).

Outcomes	Overall		*H. pylori* + at Five Months		*H. pylori*- at Five Months [b]		Persistent *H. pylori* (Positive at five Months and 11–12 years)	
	n with: *n* without Outcome	Risk Ratio (CI) *p*-Value [a]	*n* with: *n* without Outcome	Risk Ratio (CI) *p*-Value [a]	*n* with: *n* without Outcome	Risk Ratio (CI) *p*-Value [a]	*n* with: *n* without Outcome	Risk Ratio (CI) *p*-Value [a]
Overall progression in the gastric precancerous process Total *n* at risk = 295	104:191	0.98 (0.85–1.14) $p = 0.82$	91:160	1 (0.86–1.16) $p = 0.98$	13:31	1.02 (0.71–1.47) $p = 0.91$	76:140	0.97 (0.82–1.15) $p = 0.72$
Incident progression to atrophic gastritis Total *n* at risk = 98	38:60	1.01 (0.79–1.28) $p = 0.96$	33:51	1.04 (0.81–1.34) $p = 0.75$	5:9	0.67 (0.32–1.41) $p = 0.29$	28:36	1.11 (0.84–1.47) $p = 0.47$
Incident progression to intestinal metaplasia Total *n* at risk = 203	71:132	1.05 (0.88–1.25) $p = 0.57$	64:109	1.04 (0.87–1.26) $p = 0.65$	7:123	1.01 (0.63–1.63) $p = 0.97$	57:84	1.01 (0.83–1.24) $p = 0.91$
Incident progression to dysplasia or gastric cancer Total *n* at risk = 288	12:276	1.15 (0.81–1.65) $p = 0.44$	10:236	1.14 (0.77–1.67) $p = 0.52$	2:40	b	9:192	1.23 (0.86–1.78) $p = 0.26$

[a] Adjusted for age, gender, car ownership, and fruit, vegetable intake.[b] Missing RR due to sparse data.
